# Electrochemical Detection of Waterborne Bacteria Using Bi-Functional Magnetic Nanoparticle Conjugates

**DOI:** 10.3390/bios12010036

**Published:** 2022-01-12

**Authors:** Dharanivasan Gunasekaran, Yoram Gerchman, Sefi Vernick

**Affiliations:** 1 Department of Sensing, Information and Mechanization Engineering, Institute of Agricultural Engineering, Volcani Institute (ARO), Rishon leZion 5025001, Israel; dharanig@volcani.agri.gov.il; 2 The Faculty of Natural Sciences, Oranim Academic College of Education, The University of Haifa, Tivon 3600600, Israel; gerchman@research.haifa.ac.il

**Keywords:** anti-*E. coli* antibody, bi-functional MNP conjugate, ferrocene carboxylic acid, electro-chemical detection, waterborne bacterial contamination

## Abstract

Detection of microbial contamination in water is imperative to ensure water quality. We have developed an electrochemical method for the detection of *E. coli* using bi-functional magnetic nanoparticle (MNP) conjugates. The bi-functional MNP conjugates were prepared by terminal-specific conjugation of anti-*E. coli* IgG antibody and the electroactive marker ferrocene. The bi-functional MNP conjugate possesses both *E. coli*-specific binding and electroactive properties, which were studied in detail. The conjugation efficiency of ferrocene and IgG antibodies with amine-functionalized MNPs was investigated. Square-wave voltammetry enabled the detection of *E. coli* concentrations ranging from 10^1^–10^7^ cells/mL in a dose-dependent manner, as ferrocene-specific current signals were inversely dependent on *E. coli* concentrations, completely suppressed at concentrations higher than 10^7^ cells/mL. The developed electrochemical method is highly sensitive (10 cells/mL) and, coupled to magnetic separation, provides specific signals within 1h. Overall, the bi-functional conjugates serve as ideal candidates for electrochemical detection of waterborne bacteria. This approach can be applied for the detection of other bacteria and viruses.

## 1. Introduction

Concomitant with the increase in the global world population, water consumption has been increasing at an annual growth rate of 1% in the past 50 years and is projected to further increase by 20–30% by 2050 [1,2]. In contrast, the quality and availability of safe drinking water are decreasing due to domestic, industrial, agricultural, and environmental pollutions, leading to severe health hazards for humans and animals [3]. In particular, microbiological pollutants, including bacteria, viruses, and protozoa, are associated with increased diarrhea, dehydration, fever, intestinal diseases, and respiratory problems, sometimes resulting in death [4]. Remarkably, nowadays, there are 2.1 billion people without access to safe drinking water, and nearly 2 million people, primarily children, die annually due to unsafe water sources and unsafe sanitation [5,6]. Water recycling, namely treated wastewater reclamation, is becoming increasingly important as freshwater scarcity becomes prevalent worldwide. The frequency of water reuse varies globally, with Israel, Qatar, and Kuwait ranked first with >85% of their wastewater treated and reused [7]. Besides the obvious advantages, there is a growing concern for surface and groundwater contamination by pathogens originating from reclaimed wastewater. Therefore, continuous assessment of drinking and reclaimed water quality is imperative.

Detection and identification of microorganisms in water are generally carried out using conventional microbiology and molecular biology techniques, relying on membrane filtration, cell culture techniques, ELISA, PCR, microarray, staining, or microscopy examinations [8]. The above methods are considered reliable, but, especially in the bacterial context, suffer from several drawbacks, including the need for concentrating the bacteria by membrane filtration of large quantities of water, long lag times for growth (up to 72 h), the requirement for trained and highly skilled personnel and resulting costs [9,10].

Integrating biological sensing elements, conjugated nanomaterials, and electronic signal transducers on a single platform have allowed progress in diagnostic devices [11,12]. To this end, biocompatible nanomaterials such as graphene and carbon nanospheres, as well as metals such as gold, silver, and iron oxide, are widely used for the development of biosensing platforms. Among these, immuno-magnetic separation of bacteria by bioconjugate magnetic micro- or nanoparticle-antibody is adapted to overcome the abovementioned limitations [13,14]. In particular, magnetic nanoparticles (MNP) with amine functional groups exhibit high bacterial capturing efficiencies. Furthermore, the binding affinity and specificity of MNPs can be tuned by selecting specific biorecognition elements such as antibodies, or antibiotics, e.g., vancomycin and daptomycin [15]. These biofunctionalized MNPs can then be used for single-step separation and concentration of microbes from biological fluids. The detection of MNP-captured microbes is routinely carried out by a variety of analytical techniques including, surface-enhanced Raman spectroscopy (SERS), Fourier transform infrared spectroscopy (FTIR), localized surface plasmon resonance (LSPR), matrix-assisted laser desorption/ionization mass spectrometry (MALDI-MS) and bioluminescence [16]. Electrochemical methods have gained much interest due to their simple fabrication, amenability for miniaturization and biomolecule integration and, equally important, suitability for in-dependent field use by unskilled users.

Electrochemical biosensors are capable of operating as an independent unit that can be minimized, allowing direct measurement of liquid media [17,18]. The sensitivity of the electrochemical assay is inherently higher than that achieved by most detection methods due to the direct transduction of specific binding of targets to electrons without the need for photons [19]. Combining the high sensitivity, specificity and low cost of manufacturing puts electrochemical biosensors at the forefront of diagnostic devices, and developing such devices for monitoring microorganisms and pathogens in drinking water is highly desirable [20].

**Figure 1:**
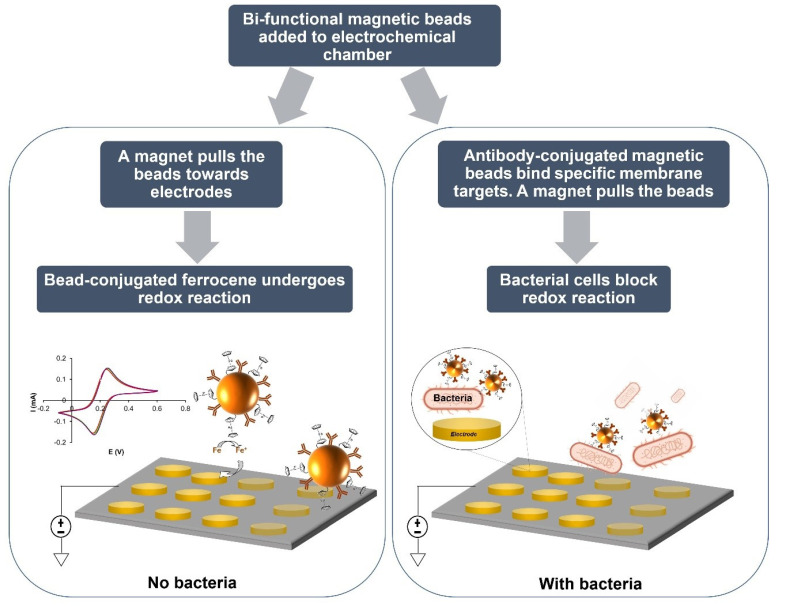
Dual-mode bioelectrochemical sensor. The measurement chamber includes a micro-fabricated electrochemical cell array (containing working, reference and auxiliary electrodes) and is equipped with a magnet. Bi-functional MNP (tagged with specific antibodies and electroactive markers, such as ferrocene) are added to the sample. Upon activation of the magnet, particles are pulled towards the electrode allowing the voltammetric detection of the ferrocene marker. In the presence of bacteria; however, antibody-labeled MNP strongly bind to membrane antigens, resulting in a decrease in the measured current response.

Methods such as impedance spectroscopy and differential pulse voltammetry have been widely used, in combination with immunomagnetic separation, for the detection of bacteria. However, coupling square wave voltammetry (SWV) with immunomagnetic separation (IMS) is less common, partly due to the absence of redox properties in both MNPs and bacteria. In this study, we aimed to develop bi-functional MNPs that contain both specific bio-recognition elements (antibodies) and redox markers (Ferrocene) for immunomagnetic separation and SWV-based detection of bacteria. The principle of operation of the device is shown in Figure 1.

## 2. Materials and Methods

MNPs (fluidMAG-Amine) 50 nm in size purchased from Chemicell Pvt. Ltd. (Berlin, Germany). Potassium hexaferrocynaide, Ferrocene carboxylic acid, N-(3-Dimethylaminopropyl)-N′-ethylcarbodiimide (EDC) and N-hydroxysuccinimide (NHS) obtained from Sigma-Aldrich, Germany. Screen-printed gold electrode obtained from BVT Technologies, Czech Republic. Anti-*E. coli* antibody—IgG and FITC-tagged Anti-*E. coli* IgG antibody were received from abcam, (Cambridge, UK). *E. coli* K12, Staphylococcus aureus (*S. aureus*-25,923), Staphylococcus epidermidis (*S. epidermidis*-42,228) and Bacillus subtilis (*B. subtilis*-6051) strains were received as a gift from Dr. Giorgi Shtenberg, ARO, Israel. Electrochemical studies were carried out using multi-channel potentiostat from Plamsens Multi EmStat (PlamsensBV, Houten, The Netherlands). Potomac yellow (PY) flourescent dye received as gift from Janelia Research Campus, Howard Hughes Medical Institute, MD, USA. UV-visible spectroscopy and fluorescence spectroscopy analysis were performed using Varioskan™ LUX (by Thermo Scientific, Waltham, MA, USA), a multimode microplate reader. FTIR anlysis carried out using Thermo Scientific Nicolet iS50 (Madison, WI, USA). Fluorescent microscopic analysis performed using fluorescent microscope (Nikon Eclipse LV150 equipped with Nikon Intensilight Epi-fluorescence illuminator) with green and red filters. Zeta potential measurements were carried out using Zetasizer nano ZS (Malvern Panalytical Ltd., Morvern, UK). All other reagents and chemicals including HEPES buffer were received from EMD Millipore Corporation, MA, USA.

### 2.1. Preparation and Characterizations of Bi-Functional Magnetic Nanoparticles (Fc-MNP-IgG)

#### 2.1.1. Preparation of Fc-MNP Conjugate

Iron oxide nanoparticles-MNPs (50 nm) with amine functional groups (1.625 × 10^13^ particles/mL) were used in all the experiments. MNPs (50 µL) were suspended in 1 mL of 1× PBS (pH 7.3) and sonicated for 15 min at RT using a bath sonicator. Ferrocene-MNP conjugates (Fc-MNP) were prepared by EDC-based conjugation method, as previously reported [21]. Ferrocene carboxylic acid (FcA-) at different concentrations (0, 4, 8, 12, 16 and 20 mM) was activated using EDC (20 mM)/NHS (100 mM) in 150 mM HEPES buffer (pH 7.3) for 2 h at RT under shaking. Then, 50 µL MNPs were added to the activated FcA- and incubated for 2 h in similar conditions. Excess unbound FcA- was removed by magnetic separation and washed with 1 mL of 1× PBS (pH 7.3) for three times. Finally, FcA-conjugation efficiency was measured by cyclic voltammetry (CV) and square wave voltammetry (SWV) analysis with a potential window of −0.2 V to +0.5 V and a scan rate of 0.1 mV/s. The redox currents measured and used to calculate the amount of Fc conjugated with MNPs.

#### 2.1.2. Preparation of IgG–MNP Conjugate

EDC-based conjugation chemistry was also used to prepare immuno-magnetic conjugates [22]. Polyclonal anti-*E. coli* IgG antibody (Abcam Cat# ab137967, specific for “O” and “K” antigenic *E. coli* serotypes) was diluted to 4000 µg/mL in sterile 1× PBS (pH 7.3) and 100 µg/mL of IgG was activated by EDC (20 mM)/NHS (100 mM) for 2 h at RT under mild shaking. The activated antibody was then mixed with 50 µL of MNPs and incubated for 2 h in similar conditions. Excess unbound antibody was removed by magnetic separation and the beads were washed three times with 1 mL of 1× PBS (pH 7.3). The amount of IgG antibody conjugated to MNPs was determined using Bradford assay.

#### 2.1.3. Preparation of Bi-Functional MNPs (Fc-MNP-IgG)

Bi-functional MNPs (Fc-MNP-IgG) were prepared by mixing of FcA- (16 mM), IgG (100 µg/mL) and MNPs (50 µL) in 1 mL of EDC (20 mM)/NHS (100 mM). The reaction mixtures were incubated for 4h at RT under mild shaking. Excess unbound FcA- and antibodies were removed by magnetic separation and washed three times with fresh 1 mL of 1× PBS (pH 7.3). Finally, the prepared Fc-MNP-IgG bi-functional conjugates were characterized by Bradford assay, CV and SWV analysis, and bacterial colony forming assay. In addition, the prepared MNP conjugates (MNPs, MNP-Fc, MNP-IgG and Fc-MNP-IgG) were further characterized by UV-visible spectroscopy, FTIR, and Zeta potential analysis (see detail in supplementary information). Moreover, bioconjugation of IgG antibody with MNPs and Fc-MNP were evaluated using FITC-tagged Anti-*E. coli* IgG antibody through Fluorescent spectroscopy and microscopy analyses (see details in supplementary information).

### 2.2. Investigation of Binding Efficiency of Mono and Bi-Functional MNP Conjugates to E. coli

Bacterial colony forming unit (CFU) assay was performed for MNPs, MNP-Fc, MNP-IgG and Fc-MNP-IgG conjugates, following standard procedure [23]. Overnight grown *E. coli* (K12 strain) cells were separated by centrifugation at 3000 RPM for 15 min and washed three times with sterile 1× PBS (pH 7.3). Washed bacterial cells (~3 × 10^9^ cells/mL) were di-luted to 10^5^ and 103 cells/mL and 100 µL of each dilution were incubated with 50 µL of MNPs conjugates for 1h at RT. After the incubation, *E. coli* attached MNPs conjugates were magnetically collected and washed three times with 1× PBS (pH 7.3). Next, the *E. coli*-MNPs conjugates were poured onto the LB agar plates and spread uniformly and the plated incubated at 37 °C overnight, and bacteria colonies counted to calculate CFU. In addition, MNP conjugates bound to *E. coli* were characterized by fluorescent microscopic analysis using the lipid binding dye Potomac yellow (PY) (see details in supplementary information).

### 2.3. Electrochemical Detection of E. coli Cells Using Bi-Functional MNP Conjugates (Fc-MNP-IgG) by SWV

The electrochemical detection of *E. coli* using Fc-MNP, IgG-MNP, Fc-MNP-IgG and MNPs was carried out by SWV. MNP conjugates (50 µL) were mixed with 100µL of *E. coli* 10^5^ cells/mL in PBS and incubated for 1h at RT. Subsequently, MNP conjugates were magnetically separated and washed three times with 200 µL 1× PBS (pH 7.3). Each MNP conjugate-*E. coli* complex (75 μL) was loaded onto the working electrode surface using magnetic forces. Voltammograms were recorded at a potential window between −0.2 V and +0.7 V at a scan rate of 25 mV/s (amplitude 0.1 V; scan increment 0.01 V; frequency 20 Hz and duration 4 s). The response currents (ΔI) were recorded and analyzed. Similarly, bi-functional MNP conjugates (Fc-MNP-IgG) were used for the detection of different concentrations of *E. coli* (0, 10^1^, 10^2^, 10^3^, 10^4^, 10^5^ and 10^7^ cells/mL) by SWV following the above procedures.

### 2.4. Fc-MNP-IgG Specificity Analysis Using Different Bacterial Strains

Specificity of Fc-MNP-IgG bi-functional conjugate towards *E. coli* K12 was studied along with different bacteria. The bacterial cultures *E. coli* K12, *S. aureus*, *S. epidermidis* and *B. subtilis* were cultured in LB medium. Overnight grown bacterial cells were separated by centrifugation at 3000 RPM for 15 min and washed three times with sterile 1× PBS (pH 7.3). Washed bacterial cells (~3 × 10^9^ cells/mL) were diluted to 10^5^ cells/mL and 100 µL of each dilution were incubated with 50 µL of Fc-MNP-IgG conjugate for 1h at RT. After the incubation, bacterial cells-attached Fc-MNP-IgG conjugate were magnetically collected and washed three times with 1× PBS (pH 7.3). Bacterial-Fc-MNP-IgG complexes were loaded and trapped on the working electrode using magnetic forces. Square wave voltammograms were recorded as previously described. The response currents (ΔI) were recorded and analyzed.

Similarly, SWV-based electrochemical detection of *E. coli* was further validated using different water samples including Milli-Q water (MQW), tap water (TW), and packaged drinking water (PDW). Fc-MNP-IgG conjugates (1 mL) were mixed with 100 mL of each water sample with and without *E. coli* K12 (100 µL of 10^7^ cell/mL). Then, the mixtures were incubated at RT for 1h under mild shaking. Subsequently, Fc-MNP-IgG were magnetically collected and washed three times with 1 mL of 1× PBS (pH 7.3). Finally, the collected bacteria-FC-MNP-IgG complexes collected from different water samples were analyzed by SWV as previously described. The response currents (ΔI) were recorded and analyzed.

## 3. Results

### 3.1. Preparation of Mono- and Bi-Functional MNP Conjugates

#### 3.1.1. Preparation of Fc-MNP Conjugates

**Figure 2:**
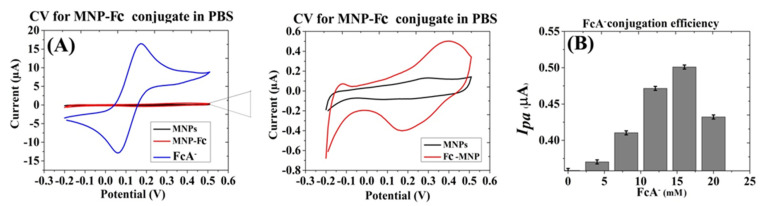
Preparation of ferrocene-conjugated MNPs. (**A**) CV of MNPs and Fc-MNP from the optimized concentration of FcA- (16 mM) in 1× PBS. (**B**) Conjugation with different concentrations of FcA- affects the measured anodic current peak. Measurements were performed in triplicates. Error bars represent the standard deviation of means for triplicate.

Electroactive MNP were prepared by conjugation with different concentrations of FcA- (0, 4, 8, 12, 16 and 20 mM) [20]. The CV analysis of ferrocene-conjugated MNP (Fc-MNP) clearly shows a reversible spectrum following ferrocene conjugation, with an anodic peak current of 0.502 µA at a potential of +0.4 V and a cathodic peak current of −0.4 µA at a potential of +0.17 V (Figure 2A right panel). Diffusion limitations are indicated by the broad current peaks area, compared with free ferrocene (Figure 2A left panel). The bar graph in Figure 2B plots the anodic current peaks recorded for Fc-MNP conjugates, exhibiting a gradual increase in anodic current with increasing concentrations of FcA-. The anodic peak current was found to saturate above 16 mM of FcA-. A Further increase of FcA- concentration adversely affects the conjugation efficiency.

Overall, the conjugation efficiency of FcA- was calculated by using the measured Fc specific redox currents obtained during SWV analyses, and it was determined at ~3%.

#### 3.1.2. Preparation of IgG-MNP Conjugates

**Figure 3:**
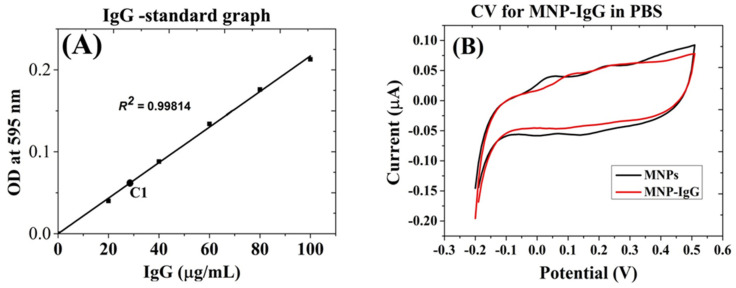
Preparation of Anti-*E. coli* IgG-MNPs conjugates. (**A**) Bradford assay standard graph for anti-*E. coli* antibody IgG (‘C1′ denotes the IgG-conjugated MNPs). (**B**) CV of MNPs and IgG-MNP conjugate in PBS.

MNPs were functionalized with anti-*E. coli* antibodies using EDC/NHS chemistry. The conjugation efficiency of IgG with MNPs was estimated and the yield was found to be 30% (~29 µg ± 2.1 of IgG/1.2 mg of MNPs), as demonstrated by the Bradford assay results shown in Figure 3A. The maximum number of IgG antibodies per MNP was estimated at ~600–963. The electrochemical characterization of IgG-conjugated MNPs is shown in Figure 3B. As expected, the obtained CV of IgG-MNP did not demonstrate any distinct redox activity but exhibited a slightly decreased non-faradic current compared with the unmodified MNPs.

#### 3.1.3. Preparation of Bi-Functional MNP Conjugate (Fc-MNP-IgG)

Bi-functional Fc-MNP-IgG conjugates were prepared as described above. The amount of IgG conjugated with MNPs was estimated by Bradford assay. The obtained results indicated that the conjugation efficiency of IgG with MNPs was reduced to 15% ± 2% (~15 µg of IgG/1.2 mg of MNPs), suggesting lesser binding of the antibody in the presence of FcA- when compared with MNP-IgG preparation.

#### 3.1.4. Characterization of MNPs Conjugates

**Figure 4:**
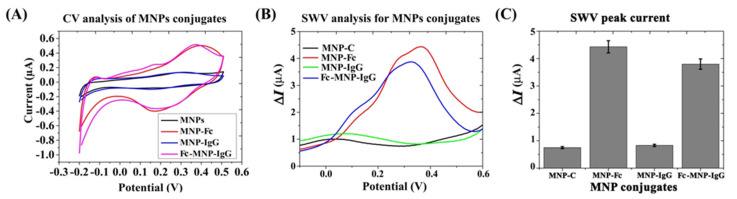
Electrochemical characterization of bi-functional MNPs (Fc-MNP-IgG) in PBS. (**A**) CV analysis of bare MNPs and conjugates. Redox peaks were found only in the Fc-MNP and Fc-MNP-IgG conjugates but not in the IgG-modified MNPs and MNPs control. (**B**) SWV analysis of MNPs conjugates. The square-wave voltammograms for MNPs control (black color), MNP-Fc conjugate (red color), MNP-IgG conjugate (green color), and bi-functional Fc-MNP-IgG conjugate (blue color). SWV parameters: amplitude 0.1 V; scan increment 0.01 V; frequency 20 Hz and duration 4 s. (**C**) The bar graph indicates peak current means for triplicate and error bars represent ±SD.

Various methods were used to characterize the prepared MNPs conjugates. First, we have assessed the yield of ferrocene conjugation to MNPs in the presence of IgG by a CV analysis. The anodic current of the bi-functional conjugate Fc-MNP-IgG was found to be higher than unmodified MNPs or IgG-MNP, as expected. As seen in Figure 4A, no significant differences were found in the conjugation efficiency of FcA- in the presence of the antibody. However, the faradic response of the ferrocene-conjugates was somewhat higher than that of the bi-functional MNP conjugate as demonstrated by the square-wave voltammograms shown in Figure 4B and C. The highest current peaks were obtained by MNP-Fc (red color) followed by the bi-functional conjugate Fc-MNP-IgG (blue color), yielding 4.4 µA at +350 mV and 3.8 µA at + 320 mV, respectively. Similar faradic current peaks were not observed in MNPs (black color) or MNP-IgG conjugate (green color) (Figure 4B,C). These results suggest that the prepared MNP-bi-functional conjugate does not result in nonspecific current signals.

The prepared MNP conjugates (MNPs, Fc-MNP, IgG-MNP and Fc-MNP-IgG) were further analyzed by UV-visible spectroscopy, FTIR spectroscopy, zeta potential measurements and fluorescence spectroscopy methods. The UV-visible absorbance spectra of MNP conjugates demonstrated an increasing intensity of the absorbance band between 200 nm–400 nm after the conjugation of MNPs with FcA-, IgG and both (FcA- and IgG) (Figure S1A). In particular, the near-UV amino acid absorbance is clearly visible following IgG conjugation. FTIR spectrum was consistent with the results obtained by UV-Vis. In particular, an intense peak in the C-N stretching mode was observed following IgG conjugation. A significant decrease in the intensity of this peak occurred following Fc conjugation (Figure S1B). Zeta potential measurements exhibited a negative potential for MNPs (−11.7 mV), which was increased significantly after functionalization with FcA- (−18.9 mV), MNP-IgG (−16.1 mV) and Fc-MNP-IgG (−16.23 mV) (Figure S1C).

Bioconjugation of IgG antibodies with MNPs and MNP-Fc was evaluated using FITC-tagged Anti-*E. coli* IgG antibody by fluorescence microscopy. FITC-IgG functionalized MNPs and MNP-Fc were prepared following the above-mentioned conjugation method. FITC specific fluorescent emission at 528 nm was found only in FITC-IgG-MNP and Fc-MNP-IgG-FITC conjugates but not in the MNPs and Fc-MNP controls (Figure S2). (See the details of MNP conjugates characterizations in the supplementary information).

### 3.2. The Binding Efficiency of Mono and Bi-Functional MNP Conjugates to E. coli

**Figure 5:**
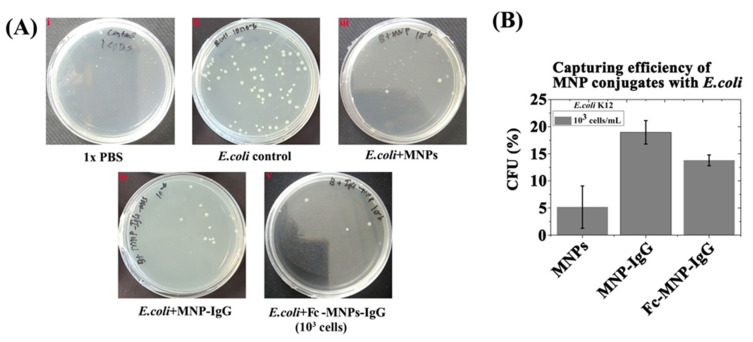
CFU assay for the assessment of binding efficiency of MNPs conjugates. (**A**) Photographic images of CFU assay for different MNPs conjugates (i) PBS, (ii) *E. coli* control (10^3^ cells/mL), (iii) *E. coli* captured by MNPs from 10^3^ cells/mL, (iv) *E. coli* captured by MNP-IgG, (v) *E. coli* (10^3^ cells/mL) captured by Fc-MNP-IgG. (**B**) Bar graph indicates the capturing efficiency of different MNPs conjugates with 10^3^ cells/mL *E. coli* as the percentage of CFU in respect to control. Error bars represent the standard deviation of means for triplicate.

The binding efficiency of mono- and bi-functional MNP conjugates to bacteria was studied by a CFU assay following immuno-magnetic separation (Figure 5). The growth of *E. coli* colonies, from a dilution of 10^3^ cells/mL, was found in all the plates except PBS control (Figure 5A—Photographic images of LB plates with *E. coli* colonies). When compared with the *E. coli* positive control, the highest percentage of CFU was obtained with MNP-IgG (~18.5% ± 2.1) followed by Fc-MNP-IgG (13% ± 1), and lastly, the unmodified MNPs (~5.1% ± 3.9). The *E. coli*-specific IgG-conjugated MNPs captured more cells compared to the bi-functional MNP conjugates (Fc-MNP-IgG), due to a higher IgG coverage. The capturing efficiency of MNP conjugates with *E. coli* cells at a concentration of 10^3^ cells/mL is summarized in Figure 5B. Interestingly, increasing the concentration of *E. coli* to 10^5^ cell/mL resulted in a decreased capturing efficiency by the bi-functional MNPs (Fc-MNP-IgG) to ~8.2% (Supplementary information Figure S3), suggesting depletion of the MNPs. In good agreement, fluorescent microscopic characterization detects more PY-stained *E. coli* captured with MNP-IgG and FC-MNP-IgG conjugates than with FC-MNP and MNPs (shown in supplementary information Figure S4).

### 3.3. Electrochemical Analysis of MNPs Conjugates Binding Efficiency to E. coli

**Figure 6:**
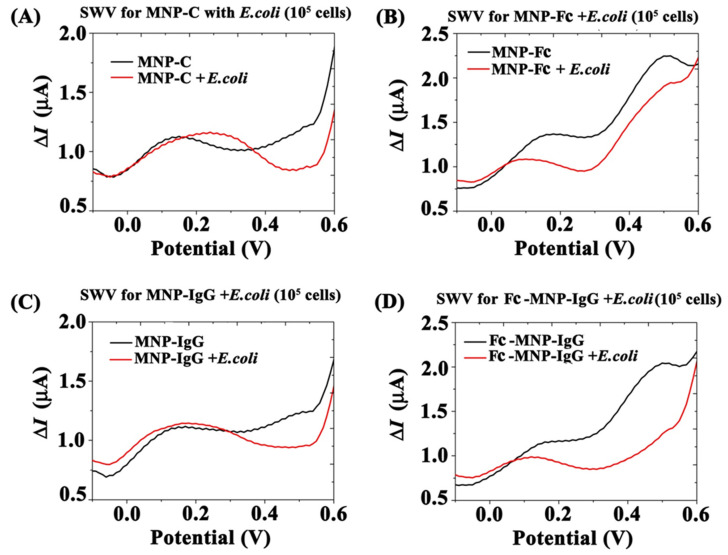
SWV detection of *E. coli* (10^5^ cell/mL) using different MNPs conjugates under a magnetic field. (**A**) SW voltammogram of MNP-C and MNP-C + *E. coli* (10^5^ cell/mL). (**B**) SW voltammogram of MNP-Fc and MNP-Fc + *E. coli* (10^5^ cell/mL). (**C**) SW voltammogram of MNP-IgG and MNP-IgG + *E. coli* (10^5^ cell/mL). (**D**) SW voltammogram of Fc-MNP-IgG and Fc- MNP-IgG + *E. coli* (10^5^ cell/mL). Black color indicates MNP conjugates without *E. coli* and red color indicates with *E. coli*. SWV parameters: amplitude 0.1 V; scan increment 0.01 V; frequency 20 Hz and duration 4s.

Following the preparation and characterization of bi-functional MNP conjugates and demonstration of their *E. coli* binding properties, the dependence of the current signals on *E. coli* binding was explored. The electrochemical behavior of immuno-magnetically separated *E. coli*-bound MNP conjugates was measured under the magnetic field. The voltammogram of MNPs control exhibited a broad current peak at Ep = +0.15 V associated with oxidation and reduction of ferrous and ferric ions of MNPs (Figure 6A, black color) [24,25]. The electroactivity of MNPs at a low potential was observed in all the MNP conjugates irrespective of reaction conditions. Following incubation of the MNPs with *E. coli*, this current peak slightly shifted from Ep = +0.15 V to Ep = +0.24 V and the oxidation current at higher potentials (>0.5 V) decreased (Figure 6A, red color), probably due to diffusion limitations following the adsorption of *E. coli* cells. A similar analysis was performed for MNP-Fc conjugates with and without incubation with bacteria (Figure 6B). The MNP-Fc voltammogram shows two current peaks, one at +0.15 V, originating from MNPs activity, as before. A second current peak appears at +0.5 V, exhibiting a high current signal (ΔI = 2.25 µA) attributed to the Fc molecules attached to the MNPs surface (Figure 6B, black color). A similar voltammetric pattern, albeit with slightly reduced current signals, is observed following incubation of the non-specific MNP-Fc with *E. coli* (Figure 6B, red color).

The voltammograms of MNP-IgG conjugates, shown in Figure 6C, demonstrate a similar pattern to that obtained by the MNPs control and are not affected by the addition of *E. coli*, as no redox agent is present. A different pattern was observed in the SWV analysis of Fc-MNP-IgG (bi-functional conjugate) with *E. coli*. As seen in Figure 6D (Black curve), the voltammogram of Fc-MNP-IgG shows the distinct Fc-specific peak at a potential +0.5 V with a current amplitude of ΔI = 2.03 µA, similar to that observed in the Fc-MNP conjugate voltammogram (Figure 6B, black curve). However, following incubation with *E. coli*, this current peak was extinguished, as seen in Figure 6D (red curve), indicating interference by the bacteria.

### 3.4. Effect of E. coli Cells Concentrations on Bi-Functional Fc-MNP-IgG Conjugates SW Voltammogram

**Figure 7:**
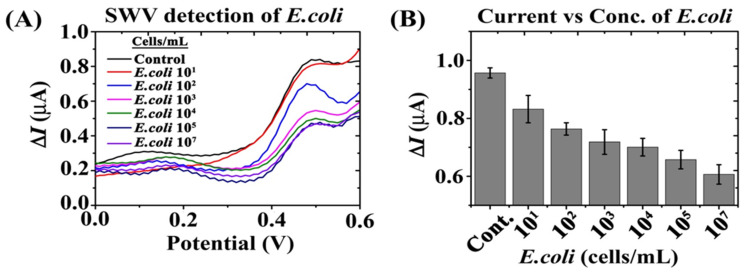
Electrochemical detection of different concentrations of *E. coli* cells using bi-functional MNPs conjugate (Fc-MNP-IgG) under magnetic field. (**A**) SW voltammogram of Fc-MNP-IgG with different concentrations *E. coli* (0, 10^1^, 10^2^, 10^3^, 10^4^, 10^5^, and 10^7^ cells/mL). (**B**) Bar graph represents the measured peak currents (ΔI) of Fc-MNP-IgG at different concentrations of *E. coli* cells. Error bars indicate the standard error of the means. SWV parameters: amplitude 0.1 V; scan increment 0.01 V; frequency 20 Hz and duration 4 s.

SWV detection was tested after incubating the bi-functional Fc-MNP-IgG conjugates with different concentrations of *E. coli* cells (0, 10^1^, 10^2^, 10^3^, 10^4^, 10^5^, and 10^7^ cells/mL) followed by magnetic separation (Figure 7). The Fc-specific current peak was observed at a potential +0.48 V, exhibiting a current amplitude of ΔI = 0.958 µA without *E. coli* (Figure 7A, Control-black color). A gradual decrease in the current was observed with increasing *E. coli* concentrations, from 10^1^ cells/mL to 10^7^ cells/mL (Figure 7A, red to violet color, respectively). The measured current signals at +0.48 V obtained at different concentrations of *E. coli* cells are presented in Figure 7B. The results demonstrate a significant dose-dependent decrease in the recorded current signal of the bi-functionalized MNPs, enabling the detection of 10 *E. coli* cells/mL.

### 3.5. Fc-MNP-IgG Specificity Analysis Using Different Bacterial Strains

**Figure 8:**
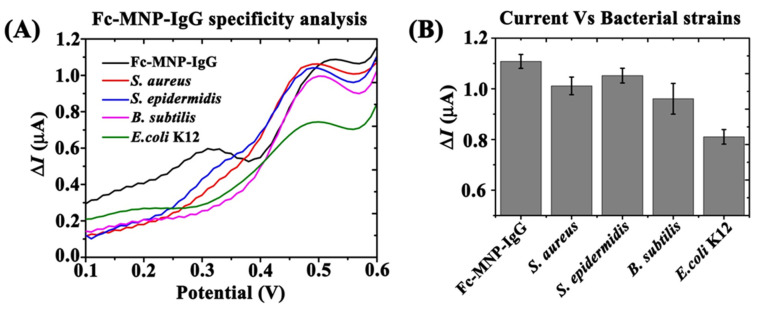
FC-MNP-IgG specificity analysis. SWV measurements were used to compare the *E. coli* K12 binding specificity with non-specific bacterial strains (*S. aureus*, *S. epidermidis* and *B. subtilis*). (**A**) SW voltammogram of Fc-MNP-IgG with different bacteria: *E. coli* K12, *S. aureus*, *S. epidermidis* and *B. subtilis* (10^5^ cells/mL). (**B**) Bar graph represents the measured peak currents (ΔI) of Fc-MNP-IgG with the different bacteria. Error bars indicate the standard error of the means. SWV parameters: amplitude 0.1 V; scan increment 0.01 V; frequency 20 Hz and duration 4s.

The specificity of bi-functional conjugate (Fc-MNP-IgG) towards *E. coli* K12 was studied with different non-specific bacterial strains (*S. aureus, S. epidermidis,* and *B. subtilis*) by monitoring the Fc-specific peak current with and without bacteria (Figure 8A). For Fc-MNP-IgG conjugate without bacteria, the recorded current at a potential +0.499 mV was found to be very high (ΔI = 1.10 µA). However, incubation with *E. coli* K12 (10^5^ cells/mL) resulted in a significant decrease in the observed current (~26.8%; ΔI = 0.81 µA). In comparison, following incubation with non-specific bacterial strains, only a limited decrease in the Fc-specific current response was observed. Non-specific binding resulted in a current signal decrease of 8.18% for *S. aureus* (ΔI = 1.01 µA), 4.5% for *S. epidermidis* (ΔI = 1.05 µA), and 11.8% for *B. subtilis* (ΔI = 0.97 µA), as shown in Figure 8B.

### 3.6. Validation in Various Water Sources

**Figure 9:**
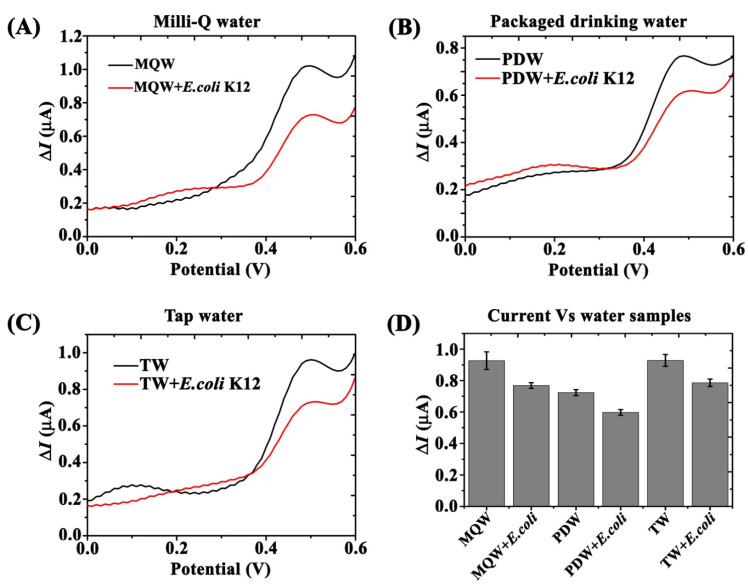
Electrochemical detection of *E. coli* K12 in different water samples using bi-functional Fc-MNP-IgG conjugate. SWV detection of *E. coli* K12 from Milli-Q water-MQW (**A**), Packaged drinking water-PDW (**B**), and Tap water-TW (**C**), with (red curves) and without (black curves) *E. coli* K12. (**D**) Bar graph indicates the measured Fc current signals from three different water samples. Error bars indicate the standard error of the means. SWV parameters: amplitude 0.1 V; scan increment 0.01 V; frequency 20 Hz and duration 4 s.

SWV based electrochemical detection of *E. coli* was further validated by using water samples from different sources (Ultra-pure MilliQ water (MQW), tap water (TW), and packaged drinking water (PDW)) with and without *E. coli* K12 (unknown concentration). The obtained voltammograms indicated the presence of bacteria in all the bacteria-spiked water samples exhibiting a similar decrease in current response (ΔI). Similar current peaks were obtained by MQW and TW samples (ΔI = 0.926 µA) at applied potential +0.499 mV. A lower current signal was observed in PDW (ΔI = 0.724 µA), as shown in Figure 9 A, B and C (black color curve). Overall, decreased Fc-specific current peaks of up to 15.5% ±2.3 were found in all the three water samples after mixing with an unknown concentration of *E. coli* K12 (Figure 9A–C—red color curve). The concentration of *E. coli* K12 in all the water samples was estimated and it was found to be ~10^2^ cells/mL. Figure 9D summarized the measured Fc current signals from all the water samples with and without *E. coli* K12.

## 4. Discussion

More than 2.2 billion people do not have access to safe drinking water [26]. Among all pollution types, microbial contaminations in water are considered a life-threatening problem, and therefore, assuring the quality of drinking and recreational water for public use is imperative. Analytical methods such as biochemical analyses, immunoassays, optical and electrochemical analyses are adapted to ensure water quality [27].

In the last two decades, electrochemical biosensors have been widely used in the area of environmental diagnostics, particularly in water quality assessment [28,29], mainly due to their many advantages, such as simplicity, high sensitivity, cost-effectiveness, portability, and ease of fabrication and miniaturization [30,31,32]. Immunomagnetic separation (IMS) with magnetic beads or MNPs is used to concentrate bacteria and other targets from the bulk solution prior to detection [23]. The development of IMS-integrated electrochemical bio-sensors is limited due to the lack of distinct electroactive properties of the MNPs. In this study, we developed an IMS-integrated SWV-based electrochemical biosensor for the detection of *E. coli* using bi-functional MNPs conjugates.

We employed the SWV technique owing to its propensity to eliminate non-faradic currents [33] that are routinely encountered by amine-terminated MNPs, as indicated by the voltammograms in Figure 2 and Figure 3 [34,35,36]. Conferring the MNPs with electroactive properties was carried out by conjugation with ferrocene (Fc), which provides an easily monitored reversible one-electron oxidation of ferrocenium, Fc ↔ Fc+ + e− [24,25]. The conjugation efficiency of Fc with MNPs was estimated at ~3.5% (Figure 2), and the Fc-specific current peaks of the labeled MNPs were clearly visible.

A similar approach was used to modify the amine surface of MNPs with a carboxylic group of IgG molecules (Figure 3) [37], yielding a terminal-specific conjugated antibody that had seemingly preserved its antigen binding affinity [38]. The bi-functional MNPs conjugates were thus prepared by conjugation with *E. coli*-specific antibody and ferrocene in a single step.

The properties of the resulting Fc-MNP-IgG bi-functional conjugates were analyzed by CV, SWV, and bacterial CFU assays. A typical Fc electroactivity was revealed, as seen in Figure 4. The conjugation efficiency of Fc was estimated at around ~3.0%, slightly lower than that obtained with the Fc-MNPs, indicating a lower density of Fc molecules in the bi-functional MNP due to the presence of IgG molecules. Additional characterizations were performed to confirm the conjugation of FcA- and IgG with MNPs. The UV-visible absorbance of MNPs was enhanced after conjugation with IgG and FC due to the changes of size, shape, and dielectric constant of MNPs and their surrounding medium [39], as well as the presence of near-UV absorbing amino acids. FTIR peak intensities clearly indicated the presence of versatile functional groups (amine, carboxyl, thiol and hydroxyl groups) on the MNPs surface after the functionalization with the antibody and the electroactive marker [40,41]. The electro-negative zeta potential of MNPs also increased dramatically after the functionalization with FcA- and IgG [42]. Finally, fluorescence emission was detected from MNP-IgG-FITC and Fc-MNP-IgG-FITC conjugates providing further support for the efficiency of our conjugation method [43].

Bacterial CFU assay was performed to investigate the binding efficiency of MNPs and the various conjugates with different concentrations of *E. coli* (10^3^ and 10^5^ cells/mL). Among these, IgG-MNP conjugate exhibited the highest binding efficiency to *E. coli* cells compared with the bi-functional Fc-MNP-IgG conjugate and MNPs control. This is likely due to the high density of IgG molecules on MNPs surface (Figure 5) [13,44].

Co-conjugation with Fc molecules in the bi-functional MNP conjugate affected the extent of IgG coverage, resulting in a lower number of IgG molecules per magnetic particle. In addition, it has been suggested that ferrocenium radicals may inhibit the growth of *E. coli* due to oxidative damage to the cell wall [45,46].

Bare MNPs (MNPs control) were found to bind very few *E. coli* cells while the prepared bi-functional conjugates bound *E. coli* cells effectively, demonstrating the formation of Fc-MNP-IgG-*E. coli* complexes. After magnetic collection, the redox activity of *E. coli*-bound Fc modified MNPs was hindered as electron transfer between the electrode surface and Fc was blocked (Figure 6). Exposure to *E. coli* cells results in a dramatic suppression in the anodic current peak, whereas no such suppression occurs with all conjugates without pre-incubation with the bacteria. In fact, incubation of *E. coli* cells with either Fc-MNP or IgG-MNP did not have any pronounced effect and yielded nearly similar voltammograms with or without bacteria. These results support the use of bi-functional MNPs conjugates as an ideal candidate for electrochemical detection of *E. coli* cells.

Finally, the bi-functional MNP conjugates were applied in the electrochemical detection of different concentrations of *E. coli*. As seen in Figure 7, Fc-specific current peak intensities were gradually decreased while increasing *E. coli* concentration from zero to 10^7^ cells/mL. The measured current signals (ΔI) were inversely proportional to *E. coli* concentrations.

The specificity of bi-functional conjugate Fc-MNP-IgG was studied with non-specific bacteria by SWV [47]. The SWV results revealed that the Fc current signal (ΔI) was significantly reduced with *E. coli* k12, but not with other bacteria, probably due to the high specificity of FC-MNP-IgG towards *E. coli* K12 [48,49]. Further validation of our detection method was provided by spiking different water samples with an unknown concentration of *E. coli* K12. The obtained voltammograms indicated the presence of bacteria in all samples. Interestingly, tap water and MilliQ water samples yielded the same background current values, whereas bottled water, which are not subjected to stringent purification processes, showed an initial decrease in the recorded current response, prior to the addition of *E. coli*, suggesting the presence of residual *E. coli* in the water. The developed method was capable of effectively detecting an estimated 100 *E. coli* cells/mL in real water samples.

*E. coli* is the most accepted indicator for water contaminants. It is a typical gram-negative, rod-shaped, cylindrical structure bacterium with the dimension of 1.0–2.1 µm long and 0.5 µm radii. [50]. With a total surface area of ~6–7 µm^2^, *E. coli* is ~100 times larger than the MNPs used in this study [51]. Thus, it can be estimated that ~140 Fc-MNP-IgG particles can bind to a single *E. coli* cell surface. The resulting complexes inherently hinder the electron transport between the electrode surface and Fc molecules during a voltammetric sweep. The inverse relationship between the measured current and *E. coli* concentrations may further be quantified. To the best of our knowledge, this is the first report on the preparation of bi-functional MNP conjugate using ferrocene electroactive marker and their application in electrochemical detection of *E. coli* cells. The sensitivity (10 CFU/mL) and detection time (1 h) of our method are better or match that of previously reported electro-chemical methods, as summarized in Supplementary Table S1. Finally, we estimate the cost per test to be highly competitive, as detailed in Supplementary Table S2.

## 5. Conclusions

We have demonstrated the feasibility of electrochemical detection of *E. coli* using bi-functional MNP conjugate (Fc-MNP-IgG) with IMS. By conferring MNPs with both dis-tinct electroactivity and specific binding affinity, we have successfully coupled immuno-magnetic separation with electrochemical detection.

The developed electrochemical method is very simple, highly sensitive (up to 10 cells/mL), cost-effective (using disposable screen-printed electrodes), and fast (detection can be completed within <1 h). This novel approach is not limited to *E. coli* detection but is also applicable for the detection of other bacteria and viruses in various water sources. These advantages are expected to ensure a diverse target market, including water treatment and management facilities, aquaculture farms and fish industries, environmental monitoring, and recreational authorities.

This new method may largely contribute to an imminent water contamination crisis by enabling intensive on-site testing. We anticipate that the outcome of this research will pave the way towards the development of a first-of-its-kind, versatile biosensing device applied in environmental diagnostics. Early detection of bacterial pathogens enables water providers and municipalities to solve problems before they turn into a crisis by identifying the source of contamination and applying timely and controlled mitigation measures such as fine-tuning of purification and filtration processes.

## Data Availability

The data presented in this study are available on request from the corresponding author.

## Supplementary Materials

The following supporting information can be downloaded at: https://www.mdpi.com/article/10.3390/bios12010036/s1, contain experimental procedures, results, and discussion of the characterization of MNP Conjugates by UV-Vis Spectroscopy, FTIR, Zeta Potential Measurements, Fluorescent Spectroscopy and Microscopy. Figure S1: Characterization of MNP conjugates (MNPs, MNP-Fc, MNP-IgG and Fc-MNP-IgG) by UV-visible spectroscopy (A), FTIR analysis (B), and Zeta potential measurements (C). Figure S2: Fluorescence analysis of FITC-IgG-MNP, FITC-IgG-MNP-Fc conjugates with MNPs and Fc-MNP controls. (A) FITC-IgG calibration curve with different concentrations (0–60 µg/mL). (B) Detection of green fluorescence from FITC-IgG-MNP, FITC-IgG-MNP-Fc conjugates with MNPs and Fc-MNP. (C) Fluorescence microscopic images for FITC-IgG-MNP, FITC-IgG-MNP-Fc conjugates with MNPs and Fc-MNP controls. Figure S3: Bacteria CFU assay for the assessment of binding efficiency of MNPs conjugates with higher concentration of *E. coli*. (A) Photographic images of CFU assay for bi-functional MNP-conjugate (Fc-MNP-IgG) with E.Coli 10^5^ cells/mL. (B) Bar graph indicates the normalized CFU for binding efficiency of bi-functional MNP-conjugate with *E. coli* 10^5^ cells/mL. Figure S4: Fluorescence microscopic images of PY-*E. coli* bounded MNP conjugates. PY-*E. coli* optical image (A) and fluorescence image (B), PY-*E. coli*-MNPs (C), PY-*E. coli*-Fc-MNP (D), PY-*E. coli*-IgG-MNP (E), PY-*E. coli*-Fc-MNP-IgG (F). Table S1: Comparison of different electrochemical methods for the detection of *E. coli*. Table S2: Estimated cost of the developed biosensor.
