# Supplementary material for: Electrochemical Detection of Waterborne Bacteria Using Bi-Functional Magnetic Nanoparticle Conjugates
Source: Biosensors (Basel). 2022 Jan 12;12(1):36. doi: 10.3390/bios12010036 (PMC8774000; doi:10.3390/bios12010036)
Supplement: Supplementary file 1 [file biosensors-12-00036-s001.zip › biosensors-1535807-supplementary.pdf]

biosensors

MDPI

Supplementary Materials: **Electrochemical Detection of Water-borne Bacteria Using Bi-Functional Magnetic Nanoparticle Conjugates**

Dharanivasan Gunasekaran [1], Yoram Gerchman [2], and Sefi Vernick [1,*]

1. Methods

1.1. .Characterization of MNP Conjugates by UV-Vis Spectroscopy, FTIR, Zeta Potential Measurements, Fluorescent Spectroscopy and Microscopy

The prepared MNP conjugates (MNPs, Fc-MNP, IgG-MNP and Fc-MNP-IgG) were dispersed in 1× PBS (pH 7.4) and 300μL each conjugates were taken for UV-Vis spectroscopy analysis using Varioskan™ LUX multimode microplate reader (Thermo Scientific, Waltham, MA, USA). The absorbance spectra were recorded from 200 800 nm and data were analyzed. Simultaneously, FTIR spectra for MNP conjugates were also recorded from 4004000 cm^{-1} using FTIR spectrophotometer (Thermo Scientific Nicolet iS50, Madison, WI, USA) by attenuated total reflection (ATR) method on the monolithic diamond crystal with 0.5 cm resolution. The collected spectra were further analyzed and peak specific functional groups were determined. Total surface charges of MNP conjugates (0.5 mL) were measured using Zetasizer nano ZS (Malvern Panalytical Ltd. UK) at room temperature with three replicate measurements.

IgG-MNPs conjugates were further studied using fluorescence microscopy with FITC tagged anti-*E. Coli* IgG antibody. Briefly, FITC-IgG antibody (100μg/mL) was used for the preparation of FITC-IgG-MNP and Fc-MNP-IgG-FITC by mixing FcA^- (16 mM), FITC- IgG (100 μg/mL) and MNPs (50μL) in 1 mL of EDC (20mM)/NHS (100mM). After the incubation, excess unbound FcA^- and antibodies were removed by magnetic separation and washed three times with fresh 1 mL of 1× PBS (pH 7.3). Fluorescence emission of MNPs, Fc-MNP, FITC-IgG-MNP and Fc-MNP-IgG-FITC were analyzed by fluorescence spectrophotometer (Varioskan™ LUX multimode microplate reader) with excitation at 493 nm and emission at 528 nm. Different concentrations of FITC-IgG ranging from 060 μg/mL were used to prepare a fluorescence calibration curve. Fluorescent images for the prepared FITC-IgG conjugated MNPs, Fc-MNP and MNPs were captured using fluorescent microscope (Nikon Eclipse LV150 equipped with Nikon Intensilight Epi- fluorescence illuminator, USA) with green filter.

1.2. Fluorescence Microscopic Characterization of E.Coli-Bound MNP Conjugates

Fluorescence assay was performed to study the binding efficiency *E. Coli* cells with MNPs, MNP-Fc, MNP-IgG and Fc-MNP-IgG conjugates [1]. Overnight grown *E. Coli* (K12 strain) cells were separated by centrifugation at 3000 RPM for 15 min and washed three times with sterile 1× PBS (pH 7.3). Separated bacterial cells (~ 3×10^9 cells/mL) were diluted to 10^5 cells/ mL in sterile 1× PBS, mixed with 10 μL (10nM) Potomac yellow (PY) fluorescent dye (dissolved in DMSO) and incubated for 30 min in dark. After the incubation, PY dye-stained *E. Coli* were separated by centrifugation at 3000 RPM for 15 min and washed three times with sterile 1× PBS. The PY-*E. Coli* 50 μL suspension was mixed with 50 μL of MNP conjugates and incubated for 1h in dark under mild shaking. Finally, PY-*E. Coli* bound MNPs conjugates were magnetically separated and washed three times with sterile water. Next, the PY-*E. Coli* bound MNPs conjugates (10 μL) were loaded onto microscopic glass slides and incubated at 37 ^{0}C for 15 min, washed with sterile water and dried with N_2. The fluorescence emission of PY-*E. Coli* bounded MNP conjugates were analyzed using fluorescent microscope (Nikon Eclipse LV150 equipped with Nikon Intensilight Epi-fluorescence illuminator, USA) with red filter and the fluorescent images were captured.

Simultaneously, PY-*E. Coli* bounded MNP conjugates fluorescence spectra were also recorded using microplate reader (Thermo Scientific™Varioskan™ LUX multimode microplate reader) with excitation at 561 nm and emission at 579600 nm. The fluorescenceintensities of PY-*E. Coli* bounded MNP conjugates were analyzed [1,2].

2. Results and Discussion

2.1. Characterizations of MNP Conjugates

2.1.1. UV-Visible Spectroscopy Analysis

The UV-visible spectra of MNPs exhibited a wide absorbance band from 200-400 nm due the spherical shape and nanometer size. Furthermore, the absorbance band intensity of MNPs increased following conjugation of Fc and more notably after IgG was conjugated (Figure S1A). The surface functionalization of electroactive marker and antibody changes the surface properties as well as dielectric constant of MNPs and its surrounding medium which affects the light absorbance properties [3,4]. Moreover, the addition of near-UV absorbing amino acids largely strongly affects the increase absorbance of the IgG conjugates. These results confirm that the MNP surface was functionalized with ferrocene and antibody molecules.

2.1.2. FTIR Analysis

Successful functionalization of electroactive marker and antibody with MNPs was further investigated by FTIR analysis, as shown in Figure S1B. Transmission peaks at 400-600 cm^{-1} were observed in all the samples, which are characteristic bands for maghemite [5]. The amide specific band was observed at 1642 cm^{-1} [6]. Also, C-N stretching vibration specific peaks were found in between 23102360 cm^{-1}. FTIR peaks between 2921 2926 cm^{-1} are characteristic features of amine and carboxyl groups interactions [7]. The broad peak at 3409 cm^{-1} corresponds to O-H stretching. These results revealed that the presence of versatile surface functional groups (amine, carboxyl, hydroxyl and thiol) on the MNPs surface [8].

2.1.3. Zeta Potential Measurements

Surface charges of MNPs, MNP-FC, IgG-MNP and Fc-MNP-IgG conjugates were determined by zeta potential measurements, as shown in the bar graph in Figure S1C. Following conjugation with FcA- a ~61% increase in MNPs zeta potential of was measured, due to the electronegativity of ferrocene carboxylic acid. a zeta potential increase of only ~38% was measured for the bi-functional Fc-MNP-IgG conjugates, reflecting the lower Fc density on the MNPs surface after the addition of IgG molecules [9,10].

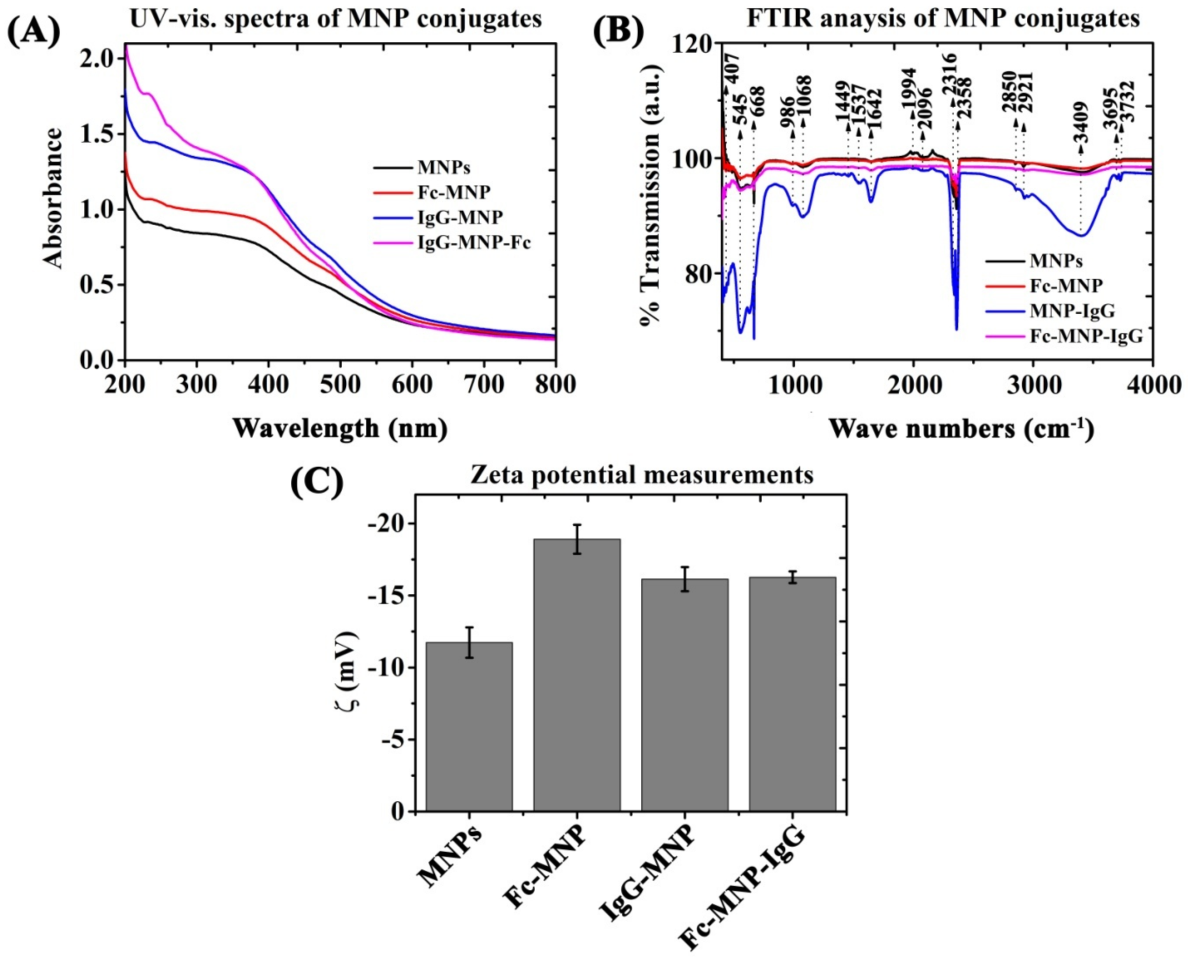

Figure S1. Characterization of MNP conjugates (MNPs, MNP-Fc, MNP-IgG and Fc-MNP-IgG) by UV-visible spectroscopy (**A**), FTIR analysis (**B**) and Zeta potential measurements (**C**). Error bars represent the standard deviation of means for triplicate.

2.1.4. Fluorescence Microscopy Analysis

The functionalization of MNPs with IgG was investigated using anti-*E. Coli* FITC-tagged IgG antibody by fluorescence microscopy. A calibration curve was prepared with different concentrations of FITC-IgG antibody from 0 60 µg /mL, resulting in a linear curve ($R^2 = 0.99$) (Figure S2A). Figure S2B bar graph illustrates the measured fluorescence emission at 528 nm from FITC-IgG-MNP and FITC-IgG-MNP-Fc conjugates with MNPs and Fc-MNP. FITC-IgG-MNP exhibited higher fluorescence than the FITC-IgG-MNP-Fc conjugate due co-conjugation of FcA-. The concentration of FITC-IgG conjugated with MNPs in both conjugates was hard to determine due to strong quenching of FITC fluorescence emission by MNPs (~142 times) [11]. Nevertheless, the fluorescence of Fc-MNP conjugate was significantly lower and MNPs control did not emit any fluorescence. The similar results were also found in fluorescence microscopy analysis with green fluorescence signal (Figure S2C). These results indicate the efficient conjugation chemistry of the mono ad bi-functional MNP conjugates.

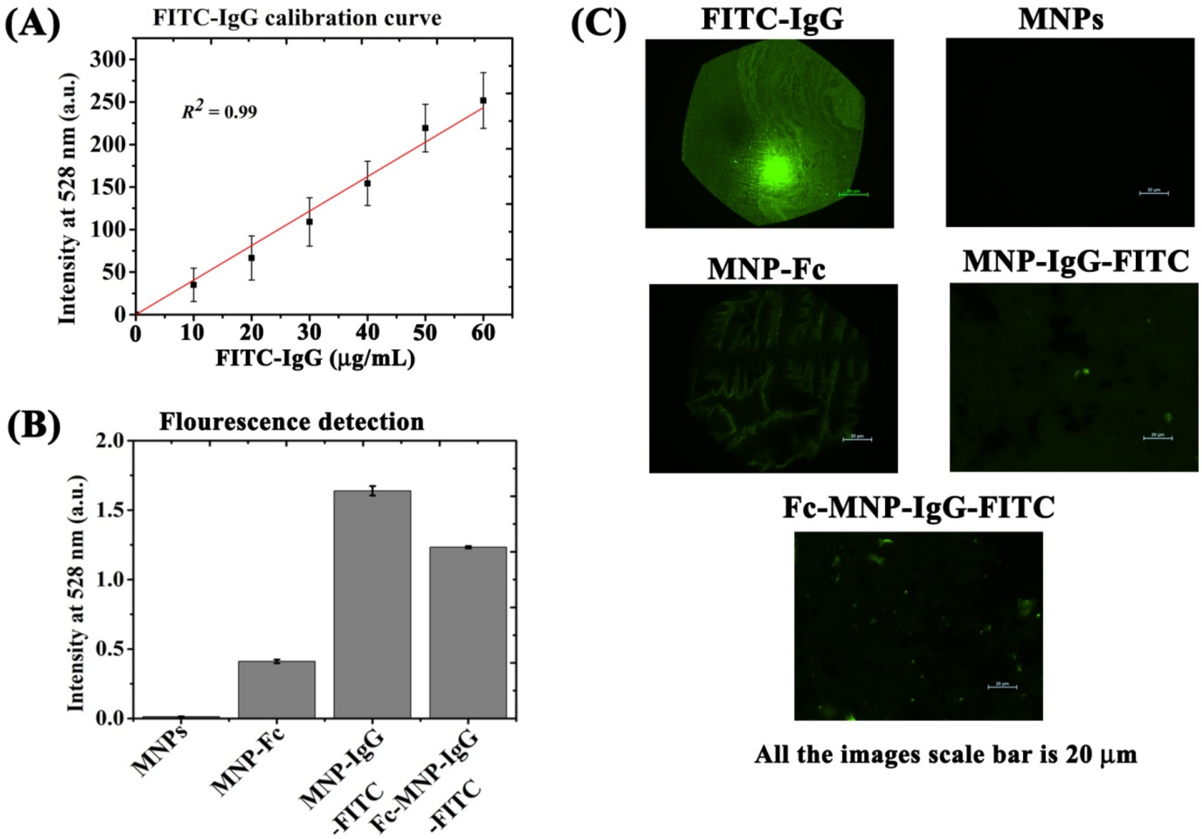

Figure S2. Fluorescence analysis of FITC-IgG-MNP, FITC-IgG-MNP-Fc conjugates with MNPs and Fc-MNP controls. (**A**) FITC-IgG calibration curve with different concentrations (0 – 60 µg /mL). (**B**) Detection of green fluorescence from FITC-IgG-MNP, FITC-IgG-MNP-Fc conjugates with MNPs and Fc-MNP. (**C**) Fluorescence microscopic images for FITC-IgG-MNP, FITC-IgG-MNP-Fc conjugates with MNPs and Fc-MNP controls. Error bars represent the standard deviation of means for triplicate.

2.2. Bacterial CFU Assay for MNP Conjugates with Higher Concentration of E.coli K12

The percentage of MNP conjugates binding efficiency was decreased at *E. coli* 10^5 cells/mL compared to lower concentration (*E. Coli* 10^3 cells/mL) (Fig. SI3). Increasing the concentration of *E. Coli* cells above 10^4 cells/ mL adversely affected the binding efficiency of MNP-IgG conjugates because large numbers of bacteria were settled down. The capturing efficiency of immunomagnetic particles depends on the ratio of Ab: MNPs, size of MNPs, and concentration of *E. Coli* cells. However, the total number of bounded *E. Coli* cells is enhanced at above the concentration of 10^5 cells /mL [12].

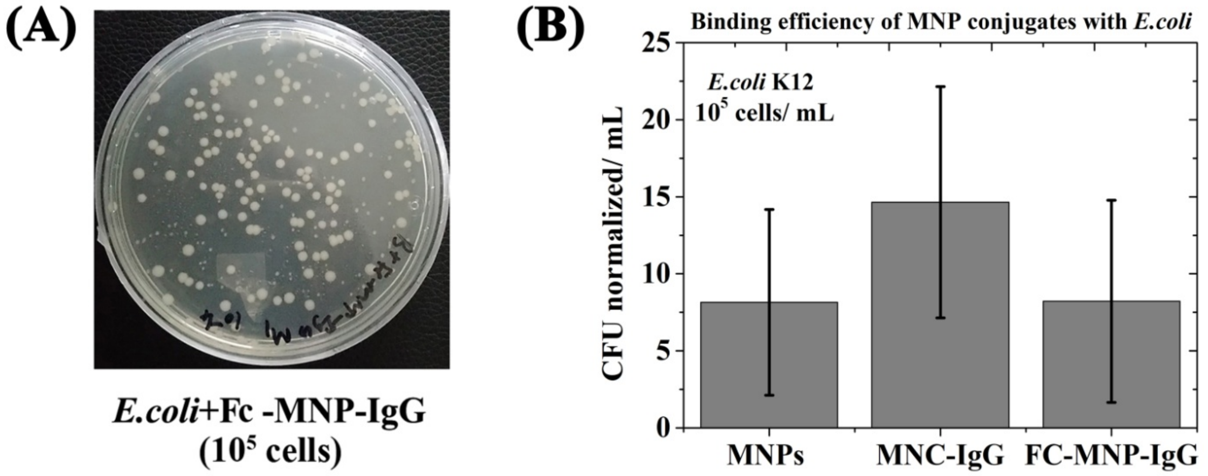

Figure S3. Bacteria CFU assay for the assessment of binding efficiency of MNPs conjugates with higher concentration of *E. Coli*. (**A**) Photographic images of CFU assay for bi-functional MNP-conjugate (Fc-MNP-IgG) with *E. Coli* 10^5 cells/mL. (**B**) Bar graph indicates the normalized CFU for binding efficiency of bi-functional MNP-conjugate with *E. Coli* 10^5 cells/mL. Error bars represent the standard deviation of means for triplicate.

2.3. Fluorescent Microscopic Characterization of E. Coli Bounded MNP Conjugates.

Potomac yellow (PY) dye [Bis(azepanyl)rhodamines- internal membrane stain] is lipid binding dye which canbind bacteria cell membrane lipids and used for high resolution imaging of cell membranesbyfluorescent microscopy analysis. Potomac yellow dye was excited at 560 nm and emission was recorded at 583 nm with a strong signal. Potomac dyes are hydrophobic and can internalize into bacterial cell wall and interact with lipid membrane through hydrophobic interactions [1,12].

Figure S4 show the fluorescent microscopic analysis of PY-stained *E. Coli* captured by different MNP conjugates (MNP-IgG, Fc-MNP-IgG, MNP-Fc and MNPs control). The rod-shaped of PY-*E.coli* cells were clearly observed under the optical microscope and green florescent laser (Figure S4 A and B, respectively. A single *E. Coli* cell is shown in inset images).Significantly higher densities of PY-*E. Coli* were observed following incubation with MNP-IgG and FC-MNP-IgG conjugates compared to MNPs control, and only a few PY-*E. Coli* cells were found with the FC-MNP conjugate (Figure S4C: PY-*E. Coli*-MNPs; D: PY-*E. Coli*-fc-MNP; E: PY-*E. Coli*-IgG-MNP, and F: PY-*E. Coli*-Fc-MNP-IgG). Binding with MNPs conjugates has resulted in agglomerations, as seen by the clusters in Figures SI4 D-F. This agglomeration caused by interactions MNP-IgG and FC-MNP-IgG with more than one PY-*E. Coli* cell. Non-specific interactions (electrostatic and ionic interactions between electronegative bacteria and positive charged MNPs) are also evident, as seen in Figure S4C. The data in Figure S4D indicates that in the case of MNP-Fc, MNPs charge was neutralized by Fc conjugation and no more interaction occurred with bacteria. This fluorescent microscopic analysis lends further support and confirms the capturing efficiency of bi-functional conjugate FC-MNP-IgG with *E. Coli*.

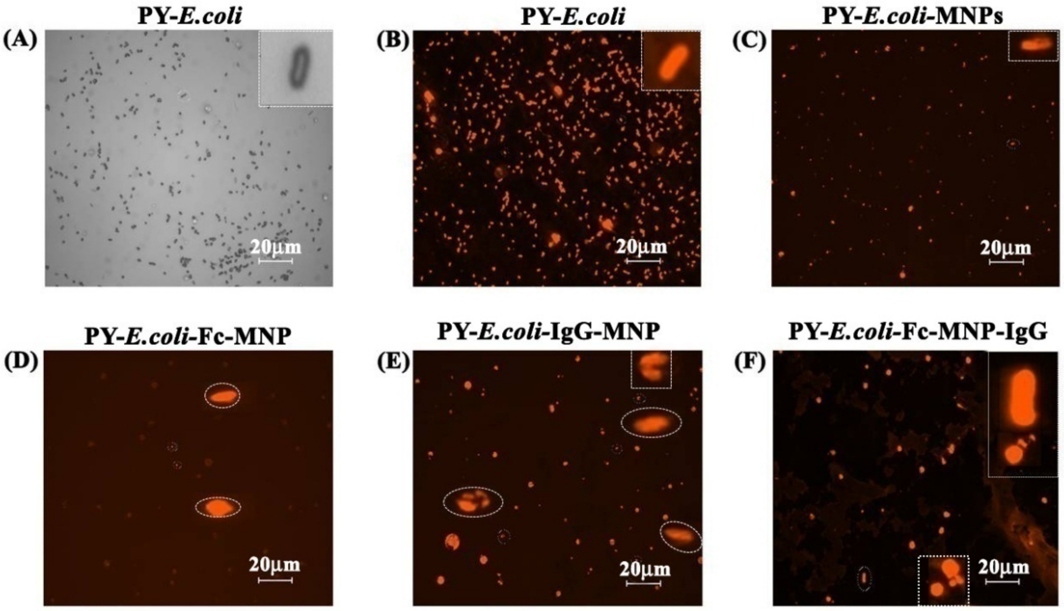

Figure S4. Fluorescence microscopic images of PY-*E. Coli* bounded MNP conjugates. PY-*E. Coli* optical image (**A**) and fluorescence image (**B**), PY-*E. Coli*-MNPs (**C**), PY-*E. Coli*-Fc-MNP (**D**), PY-*E. Coli*-IgG-MNP (**E**), PY-*E. Coli*-Fc-MNP-IgG (**F**). All the images with 20 µm scale bar.

Table S1. Comparison of different electrochemical methods for the detection of *E. Coli.*

Methods	Recognizing elements	Target	Sensitivity	Assay Time	sample	Ref.
CV	Monoclonal anti-E. Coli antibody	*E. Coli*	10^3 cfu/mL	>1h	Water	[13]
EIS	Polyclonal anti-E. Coli antibody	*E. Coli*	10 cfu/mL	>1h	Blood	[14]
EIS	DNA aptamers	*E. Coli*	10 cfu/mL	3h	Milk	[15]
CV, DPV	Monoclonal anti-E. Coli antibody	*E. Coli*	34 cfu/mL	>2h	Water and milk	[16]
DPV	DNA aptamers	*E. Coli*	80 cfu/mL	>2h	Water	[17]
SWV	Anti-*E. Coli* IgG antibody	*E. Coli* K12	10 cfu/mL	1h	Water	This work

Table S2. Estimated cost of the developed biosensor.

Component	Cost per test
Electrochemical cell chips (disposable SPE)	$ 0.8
Magnetic nanobeads (fluidMAG-amine magnetic nanoparticles)	$ 0.5
Reagents (buffer, ferrocene carboxylic acid)	$ 0.2
Antibodies (Anti-E. Coli monoclonal IgG from mouse)	$ 0.5
Complete product / (cost per test)	~ $ 2

References

1. Spahn, C.K.; Glaesmann, M.; Grimm, J.B.Ayala,A.X.;Lavis, L.D.;Heilemann, M. A toolbox for multiplexed super-resolution imaging of the *E. Coli* nucleoid and membrane using novel PAINT labels. Sci. Rep. **2018;** *8*(14768), 112.
2. Grimm, J. B.; Brown, T. A.;Tkachuk, A. N.;Lavis, L. D. General synthetic method for Si-Fluoresceins and Si-Rhodamines. ACS Cent. Sci. **2017**; *3*, 975–985.
3. Sulaiman, G. M.; Tawfeeq, A. T.; Naji, A. S. Biosynthesis, characterization of magnetic iron oxide nanoparticles and evaluations of the cytotoxicity and DNA damage of human breast carcinoma cell lines. Artif. Cells Nanomed. Biotechnol. **2018**; *46(6)*, 1215-1229.
4. Narayanan, S.; Sathy, B.N.; Mony, U.; Koyakutty, M.; Nair, S.V.; Menon, D. Biocompatible magnetite and gold nanohybrid contrast agents via green chemistry for MRI and CT bioimaging. ACS Appl. Mater. Interfaces, **2011**; *4*,251–260.
5. Tartaj, P.; Morales, M.D.; Veintemillas-Verdaguer, S.; Gonzalez-Carreno, T.; Serna C.J. The preparation of magneticnanoparticles for applications in biomedicine. J. Phys. D Appl. Phys. **2003**; *36*(13), R182–R197.
6. Asthana, A.; Verma, R.; Singh, A.K.; Susan, M.A.B.H. Glycine functionalized magnetic nanoparticle entrapped calcium alginate beads: a promising adsorbent for removal of Cu(II) ions. Chem. Eng. J. **2016;** *4*(2),1985–1995.
7. Feitoza, N.C.; Goncalves, T.D.; Mesquita, J.J.; Menegucci, J.S.; Santos, M.K.; Chaker, J.A.; Cunha, R.B.; Medeiros, A,M.; Rubim, J.C.; Sousa, M.H. Fabrication of glycine-functionalizedmaghemite nanoparticles for magnetic removal of copper from wastewater. J. Hazard. Mater. **2014;** *264*(2),153–160.
8. Wu, H.; Teng, C.; Tian, H.; Li, Y.; Wang, J. Fabrication of functional magnetic cellulose nanocomposite membranes for controlled adsorption of protein. Cellulose, **2018;** *25,* 2977–2986.
9. Yang, D..; Kroe-Barrett, R.; Singh, S.; Laue, T. IgG charge: practical and biological implications. Antibodies, **2019;** *8*, 24 118.
10. Anghelache, M.; Turtoi, M.; Petrovici, A.R.; Fifere, A.; Pinteala, M.; Calin, M. Development of dextran-coated magnetic nanoparticles loaded with protocatechuic acid for vascular inflammation therapy. Pharmaceutics, **2021;** *13*, 1414.119.
11. Ge,Y.; Zhang, Y.; He,S.; Nie, F.; Teng, G.; Gu, N. Fluorescence modified chitosan-coated magnetic nanoparticles for high-efficient cellular imaging. Nanoscale Res. Lett. **2009;** *4*,287–295.
12. Wang, Z.;Cai, R.; Gao, Z.; Yuan, Y.; Yue, T. Immunomagnetic separation: an effective pretreatment technology for isolation and enrichment in food microorganisms detection. Compr. Rev. Food Sci. Food Saf.**2020;***19*,3802–3824.
13. Setterington E. B.; Alocilja E. C. Electrochemical biosensor for rapid and sensitive detection of magnetically extracted bacterial pathogens. *Biosensors,* **2012;** *2*, 15–31.
14. Maalouf, R.; Fournierwirth, C.; Coste, J.; Chebib, H.; Saïkali, Y.; Vittori, O.; Errachid, A.; Cloarec, J.-P.; Martelet, C.; Jaffrezic-Renault, N. Label-free detection of bacteria by electrochemical impedance spectroscopy: comparison to surface plasmon resonance. *Anal. Chem.* **2007**; *79*, 4879– 4886.
15. Wang, L.; Huang, F.; Cai, G.; Yao, L.; Zhang, H.; Lin, J. An electrochemical aptasensor using coaxial capillary with magnetic nanoparticle, urease catalysis and PCB electrode for rapid and sensitive detection of *Escherichia Coli* O157:H7. Nanotheranostics, **2017**; *1*(4), 403414.
16. Guo, Y.; Wang Y.; Liu, S.; Yu, J.; Wang, H.; Cuia, M.; Huang, J. Electrochemical immunosensor assay (EIA) for sensitive detection of E. Coli O157:H7 with signal amplification on a SG–PEDOT–AuNPs electrode interface. Analyst, **2015**; *140,* 551–559.
17. Wang, H.; Zhao, Y.; Bie, S.; Suo, T.; Jia, G.; Liu, B.; Ye, R.; Li , Z. Development of an electrochemical biosensor for rapid and effective detection of pathogenic Escherichia Coli in Licorice extract. Appl. Sci. **2019;** *9*, 295, 115.
